# Observations on the Relationships between Endophytic *Metarhizium robertsii*, *Spodoptera frugiperda* (Lepidoptera: Noctuidae), and Maize

**DOI:** 10.3390/pathogens10060713

**Published:** 2021-06-07

**Authors:** Brianna Flonc, Mary Barbercheck, Imtiaz Ahmad

**Affiliations:** 1 Department of Entomology, The Pennsylvania State University, University Park, PA 16802, USA; brianna.e.flonc@usda.gov (B.F.); meb34@psu.edu (M.B.); 2 United States Department of Agriculture (USDA), Animal and Plant Health Inspection Service (APHIS)—Plant Protection and Quarantine (PPQ), Carlisle, PA 17013, USA

**Keywords:** fungal entomopathogen, endophyte, *Metarhizium robertsii*, fall armyworm, biocontrol, agricultural pest

## Abstract

Fungi in the genus *Metarhizium* are entomopathogens that can establish endophytically inside plants and benefit them through growth promotion and pest suppression. Lab- and greenhouse-based experiments were conducted to examine the effects of endophytic *M. robertsii* colonization in maize (*Zea mays*) on fall armyworm (FAW) (*Spodoptera frugiperda*). Maize seeds were inoculated with *M. robertsii* conidia, plants were evaluated for endophytic colonization, and then relative growth rate (RGR) and feeding behavior of larval FAW fed leaves from inoculated and uninoculated maize were measured. Endophytic *M. robertsii* was recovered from 60.5% of inoculated maize. In feeding bioassays, the RGR of larval FAW fed leaves of inoculated maize was no different than the RGR of larvae fed leaves from uninoculated maize. The RGR of larval FAW was positively correlated with the proportion of endophytic colonization of maize leaf and root tissues; however, in feeding assays, FAW larvae demonstrated no preference for consuming leaf tissue from inoculated or uninoculated maize. The proportion of leaf tissue consumed was unrelated to the proportion of *M. robertsii*-colonization of leaf or root tissue from source plants. We discuss possible reasons why FAW were not affected by endophytic *M. robertsii* in the context of assay methodology, FAW physiology, and induced maize defenses.

## 1. Introduction

Fall armyworm (FAW), *Spodoptera frugiperda* (J.E. Smith) (Lepidoptera: Noctuidae), is a polyphagous agricultural pest reported to feed on over 350 plant species in over 70 families, including important economic crops such as maize, tomatoes, rice, cotton, and soybeans [1,2]. Because of their high dispersal rate, voracious feeding, and ability to cause considerable economic losses, FAW is considered one of the most destructive pests of maize worldwide [3]. In many parts of Asia and Africa, FAW has the potential to transition from an invasive pest to an endemic pest where environmental conditions are favorable and hosts are available [4]. In cereals such as maize, rice, and sorghum, FAW has caused annual crop losses of up to U.S. $13 billion across sub-Saharan Africa [5]. Larval FAW has been projected to destroy over 50% of maize yields and profits, which greatly affects countries reliant on maize for more than half of their calorie and protein intake [6,7,8,9].

Management of FAW is challenging and currently relies on chemical insecticides, including carbamates, organophosphates, and pyrethroids. Heavy and indiscriminate use of various insecticides can adversely affect the ecosystem and its inhabitants, and often results in inconsistent FAW control [5,10]. Insecticide application can kill beneficial insects such as parasitoid wasps that mitigate FAW populations [11]. Transgenic *Bt*-(*Bacillus thuringiensis*) maize varieties can be used to manage FAW [12], but genetically-modified maize is not commercially available in most African countries, with the exception of South Africa [13]. Furthermore, FAW has developed resistance to both transgenic *Bt*-maize and chemical insecticides [14]. Because effective management of FAW is extremely challenging and imposes a serious threat to food security and sustainable crop productivity, an alternative or complementary management strategy is necessary.

Entomopathogenic fungi (EPF) occur naturally in soil and infect passing insects via conidia. After the host insect exoskeleton is breached by germinating conidia, hyphal bodies that obtain nutrients from the insect, including nitrogen, are formed. At the end of the infection cycle, mycelium forms, and can exit the cadaver and produce conidia or colonize plant roots and transfer N to plants in exchange for C [15]. Entomopathogenic fungi, such as those in the genus *Metarhizium* Sorokīn (Hypocreales: Clavicipitaceae), have been used as microbial control agents for pest arthropods, including several species of Noctuidae [16,17,18,19]. Susceptibility of FAW larvae and adults to direct application of *M. anisopliae* has been demonstrated in laboratory assays [20,21,22].

Although historically studied as an insect pathogen, many species of *Metarhizium* are capable of colonizing plants as endophytes [23,24]. Endophytes are plant symbionts, often bacteria or fungi, that inhabit the tissues of terrestrial plants for at least part of their life cycle without causing apparent disease symptoms [25]. Several studies have reported the establishment of endophytic *Metarhizium* in a wide-variety of plant species within root, stem, and leaf tissues [24,26,27,28,29,30,31,32]. However, some studies report that endophytic colonization by *Metarhizium* spp. occurs almost exclusively in roots and is rarely found in aboveground stem and leaf tissue, especially from field-collected plants [22,33,34,35]. Even though endophytes may be restricted to specific tissue in their plant host, their presence can have systemic effects. In our previous study, larval black cutworm, *Agrotis ipsilon* (Hufnagel), fed leaf tissue from maize plants grown from *M. robertsii*-treated seed had lower relative growth rates than black cutworm fed leaf tissue from control maize plants, and the relative growth rate of black cutworm was negatively correlated with intensity of systemic colonization [27].

Fungal entomopathogenic species and strains vary in their biological control potential, which can depend on the application method (directly to the target insect, environment, or host plant, or indirectly as an endophyte), crop species, and variety. Several authors have reported the virulence of *Metarhizium* spp. to eggs and larvae of FAW by both direct and indirect exposure. Direct application of multiple strains of *M. anisopliae* was reported to cause significant and variable mortality (up to 100%) of eggs and neonate FAW [36]. Soil application of a commercial strain of *M. anisopliae* caused 100% and 75% mortality of second and fourth instar FAW, respectively [22]. Maize sprayed with a conidial suspension of *M. rileyi* at a concentration of 1.17 × 10^4^ conidia/mL yielded a median lethal concentration (LC_50_) to FAW larvae, while a concentration of 4.03 × 10^6^ conidia/mL resulted in an LC_90_ to FAW [37]. Application of *M. anisopliae* by immersion of larvae in a spore suspension caused 67.8% mortalities of second instar FAW that were fed detached maize leaves in a laboratory assay, whereas in the field, three spray applications of the same isolate resulted in a 68% decrease in FAW infestation [21]. In a field experiment, *M. robertsii* decreased FAW incidence from 41.3% to 2.8% in the first foliar application, and 17.4% to 8.3% in the second foliar application on maize plants [38]. In laboratory assays, spray application of different strains of *M. anisopliae* caused significant but variable mortality to eggs and neonate FAW feeding on maize leaves [39]. Seed coating by microsclerotia-producing isolates of *M. robertsii*, *M. anisopliae*, and *M. humberi* indirectly reduced the survival (mortality > 55% after 7 days) of FAW in endophytically-colonized maize but did not affect the survival of *Dalbulus maidis* (Hemiptera: Cicadellidae) [40]. Tomato plants treated with *M. anisopliae* by root dipping resulted in slowed growth and reduced larval weight of second instar FAW larvae fed on leaf tissue 14 days after inoculation [41].

Here, we report the results of an experiment to determine the effects of endophytic *M. robertsii* on: (1) the growth rate of FAW larvae feeding on maize leaves grown from *M. robertsii*-inoculated seeds and (2) feeding behavior of larval FAW. We hypothesized that the growth rate of FAW larvae feeding on maize leaves grown from *M. robertsii*-inoculated seeds would be lower than those feeding on plants grown from uninoculated seeds and that endophytic colonization of maize would affect the feeding behavior of FAW in choice and no-choice feeding assays. To the best of our knowledge, this is the first study reporting results to determine the effects of *M. robertsii*-colonized maize foliage on the feeding behavior of FAW.

## 2. Materials and Methods

### 2.1. Fungal Inoculum

The isolate of *M. robertsii* (Bischoff, Rehner, and Humber) used in this experiment was isolated from soil from a field plot with winter canola (*Brassica napus* var. Wichita) [42] and baiting soil with *Galleria mellonella* larvae. Conidia were plated from cadavers on dodine-free semi-selective CTC medium [43] and initially identified by morphology. The translation elongation factor 1-alpha (TEF1-alpha) sequence used for identification was submitted to NCBI GenBank under accession number MK988559 and the single spore isolate was deposited at The Agricultural Research Service Collection of Entomopathogenic Fungal Cultures (ARSEF) under accession number 14325 [44,45]. Conidia were cultured from single-spore isolation on beads (Pro-Lab Diagnostics Microbank™ Bacterial and Fungal Preservation System) and kept at −80 °C, to be used in experiments. 

Conidia for inoculum were cultured from cryovials to CTC medium and the plates were incubated in the dark at 25 ± 2 °C. Under aseptic conditions, conidia were harvested and suspended in sterile 0.05% aqueous solution (*v*/*v*) of Triton™ X-100 (Dow Chemical Co., Midland, MI, USA). The suspension was shaken vigorously for one minute for homogenization, then filtered through four layers of sterile cheese cloth to remove mycelium fragments from the suspension. The concentration of the suspension was determined using a Neubauer hemocytometer and examining conidia under a compound microscope at 400× magnification. The conidial suspension was diluted to a concentration of 1 × 10^8^ conidia mL^−1^ for seed inoculation. Conidial viability was assessed by plating 100 µL of the suspension on Sabouraud dextrose agar (SDA) medium and counting how many conidia germinated or had visible germ tubes twice the length of the conidium in a randomly selected field of view of the compound microscope at 400× magnification. Only conidial suspensions with a germination rate >90% were used in experiments.

### 2.2. Seed Surface Sterilization and Inoculation

Prior to inoculating maize, seeds were surface sterilized (*Zea mays* var. Master’s Choice 4050, organic) under aseptic condition by immersion in 0.5% sodium hypochlorite for two minutes and 70% ethanol for two minutes, then the seeds were serially rinsed three times in sterile distilled water [46]. A small volume of the final rinse water (100 µL) was plated onto 100 × 65 mm Petri dishes containing SDA and incubated in the darkness at 25 ± 2 °C for 10 days to determine if surface sterilization was effective in removing microbes. Surface-sterilized seeds were placed in 100 mL of conidial suspension (1 × 10^8^ conidia mL^−1^) in a 250 mL sterile beaker covered with aluminum foil. Control seeds were placed in a 250 mL beaker containing 100 mL of 0.05% Triton X-100 aqueous solution, also covered with aluminum foil. Both beakers were placed on a shaker plate at 10 rpm for 2 h, then the seeds were planted directly from the beakers using sterile spatulas.

### 2.3. Plant Growth Medium

Field soil and potting medium (Vigoro Industries, Inc., Northbrook, IL, USA) were mixed in a 1:1 ratio (*v*/*v*) and steamed twice at ~121 °C for 2 h. The soil mixture rested for 48 h to avoid toxicity to plants from increased soluble salt, ammonium nitrogen, and manganese [47]. One conidia-inoculated maize seed or one control seed was planted in each steamed pot (15 cm diameter × 14.7 cm tall plastic pot) at a depth of ~2.5 cm using separate sterile spatulas for each treatment. Pots were placed in a random design in a greenhouse (16L/8D photoperiod at 25 ± 2 °C) and watered as needed.

### 2.4. Evaluation of Endophytic Colonization of Maize

**Figure 1:**
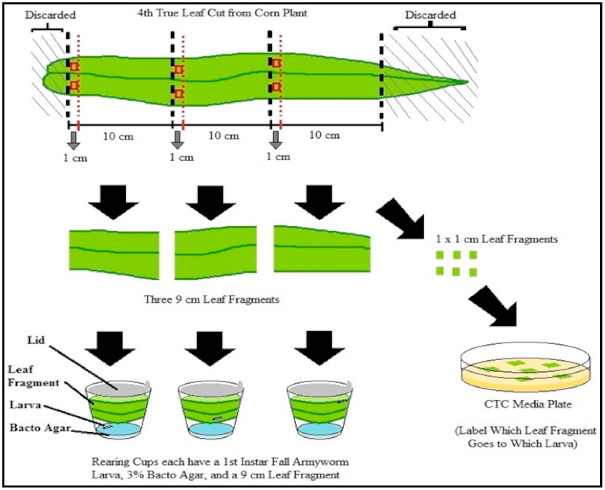
Design of the detached-leaf feeding assay to determine relative growth rate of fall armyworm (FAW). The same leaf used for first instar FAW larvae feeding was used to evaluate endophytic colonization. From each leaf, six 1 × 1 cm leaf sections were generated to evaluate endophytic colonization by *M. robertsii* and three 9 cm long leaf segments were used for the FAW feeding assay.

Endophytic colonization of *M. robertsii* was assessed in maize plants at the V4 stage. The fourth true leaf from each plant was cut into three 10 cm long sections, then a 1 cm wide cross-section was excised from each section to determine the presence or absence of the endophyte (Figure 1). Two root sections were excised from each plant, one from the distal end of the seminal root and a second from approximately 1 cm below the mesocotyl. The leaf and root sections were surface sterilized by immersion in 0.5% sodium hypochlorite for two minutes, 70% ethanol for two minutes, and then serially rinsed three times in sterile deionized water under a laminar flow hood. To determine the success of the surface sterilization, 100 µL of the final rinse water was plated onto SDA medium and checked after 10 days. Sterile dissecting scissors were used to cut away the outer edges of the leaf segments and the ends of the root segments to remove dead cells. Each leaf section was cut into two, 1 × 1 cm sections and the resulting six sections were plated in a labeled Petri dish prepared with CTC medium and pressed flat against the surface of the medium. The plates were sealed with parafilm and incubated in the dark at 25 ± 2 °C for 14 days. *M. robertsii* was identified by its distinctive white hyphal growth and dark green conidia. Fungal growth that emerged from the plant sections was cultured and identified as *M. robertsii* using the aforementioned sequencing [45].

Inoculated maize plants were separated into two categories: “Inoculated and Detected” in which *M. robertsii* was recovered from plant tissue and “Inoculated and Not Detected” in which *M. robertsii* was not recovered from plant tissue. The endophytic colonization of *M. robertsii* was considered successful if the inoculated maize grew *M. robertsii* from at least one root or leaf section. The proportion of endophytically colonized leaf and root tissue was recorded from the total sections plated.

### 2.5. Fall Armyworm 

FAW eggs were obtained from Benzon Research (Carlisle, PA, USA) and placed in a 500 mL deli container in an incubator at 25 ± 2 °C (16L/8D) until they hatched. Upon hatching, neonate larvae were individually transferred to corn earworm (*Helicoverpa zea*) artificial diet [48,49] in 30 mL plastic rearing cups (Frontier Agricultural Sciences, Newark, DE, USA) using a paintbrush and held until they were used in assays. Each larva was provided with a 1 × 1 cm cube of diet that was replaced as needed. Instar was determined by recording head capsule molts and removing cast exuviae from the rearing cups.

### 2.6. Relative Growth Rate of Fall Armyworm 

A detached-leaf feeding assay was performed to determine the effect of endophytic *M. robertsii* on the relative growth rate of first instar FAW [50]. As first instar larvae were too small to be weighed accurately as individuals and therefore larvae were weighed in groups of 30 and the total weight divided by 30 to determine the individual average weights. After weighing, larvae were placed individually in plastic cups with 3 mL of 3% Bacto^®^ agar (BD Difco, Franklin Lakes, NJ, USA) at the base of the cups to prevent desiccation.

To construct the assay arenas, the fourth true leaf was harvested from treated and control maize plants and the ends of the leaves were trimmed to obtain 30 cm long sections. The 30 cm length of leaf was cut into three equal 10 cm long sections. A 1 cm section from each 10 cm long leaf section was excised to use for evaluation of endophytic colonization (Figure 1). A 9 cm leaf section was placed in each rearing cup with a pre-weighed larva and allowed to feed at 27 ± 2 °C in 16L/8D photoperiod for 96 h. After 96 h, each larva was weighed individually to obtain a final weight and calculate their relative growth rate [51].

### 2.7. Feeding Behavior Assay and Experimental Design

A laboratory feeding assay with third instar FAW was conducted to determine if endophytic *M. robertsii* affects the amount of maize tissue consumed by FAW larvae [52]. Surface disinfected leaf and root tissues from each plant were plated to detect endophytic colonization by *M. robertsii*. The assay arena consisted of a sterile Petri dish (100 mm diameter) with autoclaved Whatman^®^ filter paper No. 1 moistened with 2 mL of sterile water to prevent desiccation of the larvae. Three types of assay arenas were prepared: (1) a no-choice assay with six leaf squares from control plants, (2) a no-choice assay with six leaf squares from *M. robertsii*-inoculated plants, and (3) a choice assay with three control leaf squares alternating with three leaf squares from *M. robertsii*-inoculated plants.

**Figure 2:**
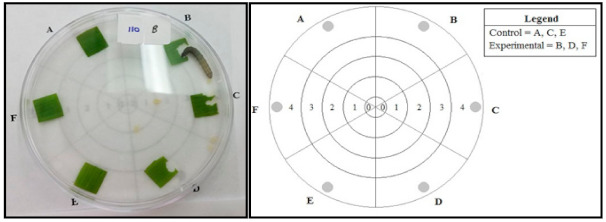
Assay arena for the detached leaf feeding preference experiment using third instar FAW. Shaded circles next to the letters A, B, C, D, E, and F indicate locations of leaf squares. In the no-choice assays, all six leaf squares from control or from the *M. robertsii*-inoculated plants were placed at locations A, B, C, D, E, and F. In the choice assay, the leaf squares from control plants were placed at A, C, and E and those from *M. robertsii*-inoculated plants were placed at B, D, and F.

To prepare assay arenas, leaf squares were excised from the fourth true leaf of treated and untreated maize plants with a 1.1 × 1.1 cm hole-punch. Each arena was placed on top of a diagram with labeled zones so that the location of the FAW larvae could be recorded at different time intervals (Figure 2). The leaf squares were arranged around the edge of the Petri dish and each leaf square was assigned a letter from A to F. In the no-choice assays, all six leaf squares from control plants or from the *M. robertsii*-inoculated plants were placed at locations A, B, C, D, E, and F. In the choice assay, three leaf squares from control plants were placed at locations A, C, and E and three leaf squares from *M. robertsii*-inoculated plants were placed at locations B, D, and F so that the leaf squares alternated around the perimeter. The Petri dishes were closed immediately after placing the FAW larvae inside to limit the effects of external stimuli.

To conduct the assay, one third instar larva was placed in the center of the prepared plate and its locations were recorded as it moved using zones formed by concentric circles 1 cm apart, representing the distance the larva traveled from the original center circle [52]. The larvae were not starved prior to the experiment. The orientation of the individual arena diagrams was randomized and recorded to avoid bias from external stimuli. The location of each FAW larvae was recorded at 30, 60, 90, and 120 min using the zones to indicate whether larva had a tendency to avoid or prefer leaf squares from different treatments. At the end of the assay, all leaf squares were collected and attached to a sheet of paper with clear tape to obtain a scanned image. We used ImageJ (https://imagej.nih.gov/ij/, accessed on 21 September 2017) to measure the area consumed from each leaf square.

Three replicated feeding trials were performed. In Trial 1, four control plants and seven *M. robertsii*-inoculated plants were used to make 50 assay arenas comprised of 10 no-choice arenas with only control leaf squares, 10 no-choice arenas with only leaf tissue from *M. robertsii*-inoculated plants, and 30 choice arenas with tissue from both control and treated leaf squares. In Trial 2, 9 control plants and 10 *M. robertsii*-inoculated plants were used to make 50 assay arenas comprising of 10 no-choice control arenas, 10 no-choice treatment arenas, and 30 choice arenas. In Trial 3, 10 control plants and 20 *M. robertsii*-inoculated plants were used for 55 arenas divided into 10 no-choice control arenas, 20 no-choice treatment arenas, and 25 choice arenas.

### 2.8. Statistical Analyses

All statistical analyses were carried out in JMP^®^ Pro 15.0 (SAS Institute Inc.; Cary, NC, USA) unless stated otherwise. Mixed model ANOVA was used to determine the effects of treatments on relative growth rate and feeding preference of FAW. All treatment variables were designated as fixed factors and block (trial replicate number) as a random factor. When the model was significant, the Tukey’s honest significant difference post-hoc test of means was used. The results of analyses were considered significant at *p* < 0.05. Regression analyses were conducted to assess the relationship between the proportion of *M. robertsii*-colonized root and leaf tissues per plant and insect growth parameters, using the proportion of colonized tissues as the explanatory variable and insect performance measures as the response variables. For all analyses, the proportions were transformed using square root arcsine transformation to meet assumptions of normality, equality of variances, and to reduce heterogeneity of variances [53]. Data presented in figures are not transformed.

## 3. Results

### 3.1. Evaluation of Endophytic Colonization

Endophytic *M. robertsii* was recovered from the tissue of 201 plants out of 295 *M. robertsii*-inoculated plants with a mean recovery of 60.5 ± 10.6%. Of the 295 *M. robertsii*-inoculated plants, *M. robertsii* was recovered from only the roots of 42 plants (12.0 ± 2.70%), only the leaves of 65 plants (14.0 ± 6.2%), and from both roots and leaves of 94 plants (21.7 ± 9.71%). Endophytic *M. robertsii* was not recovered from 94 plants (39.5 ± 10.6%). No *M. robertsii* was detected in any tissue among the uninoculated plants.

### 3.2. Relative Growth Rate of Fall Armyworm

There was no difference (F_2,215_ = 0.70; *p* = 0.49; n = 219) in the relative growth rate of FAW among Inoculated and Detected (0.46 ± 0.003 mg/day), Inoculated and Not Detected (0.39 ± 0.01 mg/day), and Not Inoculated (0.42 ± 0.001 mg/day) plants after feeding on maize leaf tissue for 96 h.

**Figure 3:**
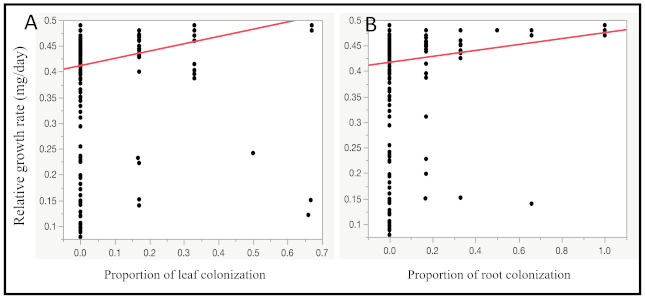
Correlation between relative growth rate of fall armyworm larvae (**A**) with proportion of leaf colonization (R^2^_adj_ = 0.07, *p* = 0.002, Est. = 0.14); and (**B**) with proportion of root colonization (R^2^_adj_ = 0.04, *p* = 0.02, Est. = 0.06) by endophytic *M. robertsii*.

The relative growth rate of FAW larvae was positively, but weakly, correlated with intensity of leaf infection (R^2^_adj_ = 0.07, *p* = 0.002, Est. = 0.14, n = 130; Figure 3A) and intensity of root infection (R^2^_adj_ = 0.04, *p* = 0.02, Est. = 0.06, n = 130; Figure 3B) after feeding on maize leaf tissue for 96 h.

### 3.3. Maize Leaf Consumption by Fall Armyworm

In choice assays, endophytic colonization by *M. robertsii* had no effect (F_2,50_ = 0.48; *p* = 0.62; n = 55) on the amount of maize leaf tissue consumed by FAW larvae or larval mortality. The percentage of leaf tissue consumed by FAW after 2 h was 32.0 ± 4.0%, 27.3 ± 5.4%, and 26.4 ± 4.3% in the Inoculated and Detected, Inoculated and Not Detected, and Not Inoculated groups, respectively. In choice assays, the amount of maize leaf tissue consumed after 2 h by FAW was not correlated with intensity of leaf infection (R^2^_adj_ = 0.07, *p* = 0.16, Est. = 0.48, n = 30; data not shown) nor with intensity of root infection (R^2^_adj_ = −0.03, *p* = 0.88, Est. = 0.03, n = 30; data not shown).

In no-choice assays, there was no difference (F_2,50_ = 0.67; *p* = 0.52; n = 55) in the amount of maize leaf tissue consumed by FAW after 2 h among the Inoculated and Detected (25.2 ± 4.9%), Inoculated and Not Detected (28.3 ± 6.8%), and Not Inoculated (31.2 ± 5.4%) groups. The amount of leaf tissue consumed by FAW in 2 h was not correlated with intensity of leaf infection (R^2^_adj_ = −0.01, *p* = 0.39, Est. = 0.25, n = 35; data not shown) nor with intensity of root infection (R^2^_adj_ = −0.02, *p* = 0.54, Est. = −0.13, n = 35; data not shown) of the source plants.

## 4. Discussion

Soilborne entomopathogenic fungi (EPF), including *Metarhizium* spp., have been the subject of intensive research since the discovery of their benefits to plants while residing in the rhizosphere or as endophytes [24,54]. Overall, 60.5% of maize plants grown from inoculated seed were colonized by endophytic *M.* *robertsii* conidia, making seed inoculation a fairly successful method of inoculation. The concurrent colonization of root and leaf tissue is indicative of systemic establishment. Our results are consistent with studies reporting endophytic *Metarhizium* in stem, leaf, and root tissue [24,26,27,28,29,30,31,32]. Many studies only recovered *Metarhizium* spp. from plant roots [22,33,34,35,55,56], most likely because roots are the first source of conidial contact.

We hypothesized that the relative growth rate of FAW larvae fed *M.* *robertsii*-inoculated maize leaves would be lower than the relative growth rate of FAW larvae fed control maize leaves. We also hypothesized that successful endophytic colonization would affect the amount of leaf tissue consumed by FAW. The presence of endophytic *M. robertsii* in maize leaf tissue did not affect the growth or feeding behavior of FAW larvae. Therefore, our hypotheses were not supported. There was also no detectable difference in the relative growth rate of first instar FAW larvae fed leaf tissue from Inoculated and Detected, Inoculated and Not Detected, or control maize plants. However, the relative growth rate was positively, but weakly, correlated with the intensity of leaf and root colonization, as determined by the number of tissue sections per plant from which *M. robertsii* was re-isolated.

The interactions among different host plants, insects, and fungal entomopathogens at specific and subspecific levels is complex. The weak positive relationship between endophytic *M. robertsii* presence and relative growth rate of FAW was inconsistent with similar studies that demonstrated negative effects of endophytic *Metarhizium* spp. on plant-feeding insects [38,41,57,58]. In our previous study, the relative growth rate of larvae of another noctuid species, black cutworm (*Agrotis ipsilon*), was lower after feeding on leaf tissue from *M. robertsii*-inoculated maize compared with the relative growth rate of black cutworm feeding on control leaf tissue, and unaffected by colonization intensity [27]. In our current study, *M. robertsii*-colonized leaf tissue may have had no effect on FAW relative growth rate because of larval resistance to maize defense toxins and proteins. FAW is a generalist herbivore capable of enzymatically detoxifying toxic compounds, such as benzoxazinoids, a defensive compound common in maize [59], and plant protease inhibitors [60,61,62,63]. Colonized maize tissue may also have had defensive metabolites that were ineffective against FAW owing to the deactivation of metabolites in their midguts via antimicrobial peptides and high alkalinity, and the rapid evacuation of harmful metabolites from their bodies [27,64,65,66]. Plant defense interactions vary depending on which plant cultivar, herbivore species, and endophytic isolate is used in experiments [2,22,36,39,41].

Owing to the high cost to the plant of mobilizing defense pathways, the quality of the plant as a food resource may also be altered by endophytic colonization, which may affect the feeding rate of FAW [67,68] or the colonization of the plant by *M. robertsii* [69]. Similar to some phytopathogenic fungi, some endophytic fungi can elicit the jasmonic acid (JA) pathway and activate the synthesis of benzoxazinoids in the host plant [70,71,72]. If the plant defense pathway is already activated by endophytic fungi, herbivory may not elicit an additional defense response in the plant owing to antagonistic crosstalk in the signaling pathways [73,74]. The suppressive effects of *Metarhizium* spp. on FAW growth reported in other studies may be due to relatively higher level of activation of plant defense pathways due, in part, to differences associated with crop variety and fungal isolate. In a previous experiment, inoculation of maize with *M. robertsii* was found to alter gene expression in the JA and SA biosynthesis pathways, which proactively increased maize plant defense against herbivores [27]. However, crosstalk of signal pathways due to endophytic colonization can vary by plant, endophyte isolate, and insect species [75,76,77,78,79].

In our experiment, the lack of growth change, feeding preference, or mortality of FAW in response to endophytic *M. robertsii* in maize indicates that FAW were not affected by any toxins produced or mediated by endophytic *M. robertsii*. Although FAW are susceptible to direct infection by *Metarhizium* [80], exposure to endophytic *M. robertsii* in the maize plants used in our assays does not appear to adversely affect them. Excised maize leaf tissue was reported to emit higher defense sesquiterpenes compared with intact maize plants [80,81] owing to plant injury mimicking herbivory; however, additional cues of herbivory, such as fatty acid-amino acid conjugates, frass, and oral secretions at the injury site of the plant, are usually needed to elicit plant defense response [50,82,83,84,85]. Direct defense mechanisms can impact larvae by making the plant tissue toxic or repellent to the herbivore through secondary metabolites, volatile blends, or physical structures on the plant that impede the ability of herbivores to feed [83]. Although the design of our assay arenas contained leaf squares from multiple treatments that could cause signal crossover, a study with a similar design to determine feeding preference of larval diamondback moth (*Plutella xylostella* L.) reported no signal crossover [52].

In summary, through seed inoculation, we were able to establish endophytic colonization of root and foliar tissue of maize by *M. robertsii*. Seed inoculation with *M. robertsii* did not affect the relative growth rate or food choice of FAW larvae. Further research using molecular analysis, such as quantitative RT-PCR, would help to determine the level and location of endophytic *M. robertsii* colonization in maize [86] and aid in understanding how the intensity of endophytic colonization could impact the feeding behavior of a range of chewing insects. Replication of this experiment with intact plants could provide information about altered plant volatile and induced defense compounds that may differ from detached tissues [87].

## Data Availability

The data presented in this study are available on request from the corresponding author.

## References

[1] Barlow V.M., Kuhar T.P. (2009). Fall armyworm in vegetable crops. Va. Coop. Ext..

[2] Montezano D., Specht A., Sosa-Gómez D., Roque-Specht V., Sousa-Silva J., Paula-Moraes S., Peterson J., Hunt T. (2018). Host plants of *Spodoptera frugiperda* (Lepidoptera: Noctuidae) in the Americas. Afr. Entomol..

[3] Zacarias D.A. (2020). Global bioclimatic suitability for the fall armyworm, *Spodoptera frugiperda* (Lepidoptera: Noctuidae), and potential co-occurrence with major host crops under climate change scenarios. Clim. Chang..

[4] FAO, CABI (2019). Community-based fall armyworm (Spodoptera Frugiperda) monitoring, early warning and management.

[5] Harrison R.D., Thierfelder C., Baudron F., Chinwada P., Midega C., Schaffner U., Berg J.V.D. (2019). Agro-ecological options for fall armyworm (*Spodoptera frugiperda* JE Smith) management: Providing low-cost, smallholder friendly solutions to an invasive pest. J. Environ. Manag..

[6] CABI New report reveals cost of fall armyworm to farmers in Africa, provides recommendations for control. https://www.cabi.org/news-article/new-report-reveals-cost-of-fall-armyworm-to-farmers-in-africa-provides-recommendations-for-control/.

[7] Day R., Abrahams P., Bateman M., Beale T., Clottey V., Cock M., Colmenarez Y., Corniani N., Early R., Godwin J. (2017). Fall armyworm: Impacts and implications for Africa. Outlooks Pest Manag..

[8] Macauley H. Cereal crops: Rice, maize, millet, sorghum, wheat: Background paper. Proceedings of the Conference on Feeding Africa.

[9] Rwomushana I., Bateman M., Beale T., Beseh P., Cameron K., Chiluba M., Clottey V., Davis T., Day R., Early R. (2018). Fall armyworm: Impacts and implications for Africa.

[10] Kelly P. (2009). ‘Monkey’ Business. Locke Stud..

[11] De Lange E.S., Farnier K., Degen T., Gaudillat B., Aguilar-Romero R., Bahena-Juárez F., Oyama K., Turlings T.C.J. (2018). Parasitic wasps can reduce mortality of teosinte plants infested with fall armyworm: Support for a defensive function of herbivore-induced plant volatiles. Front. Ecol. Evol..

[12] Siebert M.W., Tindall K.V., Leonard B.R., Van Duyn J.W., Babcock J.M. (2008). Evaluation of corn hybrids expressing CrylF (Herculex^®^ I Insect Protection) against fall armyworm (Lepidoptera: Noctuidae) in the southern United States. J. Entomol. Sci..

[13] Prasanna B., Huesing J.E., Eddy R., Peschke V.M. (2018). Fall armyworm in Africa: A guide for integrated pest management.

[14] Ingber D., Mason C., Flexner L. (2018). Cry1 Bt susceptibilities of fall armyworm (Lepidoptera: Noctuidae) host strains. J. Econ. Entomol..

[15] Behie S.W., Zelisko P.M., Bidochka M.J. (2012). Endophytic insect-parasitic fungi translocate nitrogen directly from insects to plants. Science.

[16] Ahmad I., Jiménez-Gasco M.D.M., Luthe D.S., Barbercheck M.E. (2020). Systemic colonization by *Metarhizium robertsii* enhances cover crop growth. J. Fungi.

[17] Ahmad I., Zaib S. (2020). Mighty microbes: Plant growth promoting microbes in soil health and sustainable agriculture.

[18] de Faria M.R., Wraight S.P. (2007). Mycoinsecticides and mycoacaricides: A comprehensive list with worldwide coverage and international classification of formulation types. Biol. Control..

[19] Lacey L., Grzywacz D., Shapiro-Ilan D., Frutos R., Brownbridge M., Goettel M. (2015). Insect pathogens as biological control agents: Back to the future. J. Invertebr. Pathol..

[20] Gutiérrez-Cárdenas O.G., Cortez-Madrigal H., Malo E.A., Gómez-Ruíz J., Nord R. (2019). Physiological and pathogenical characterization of *Beauveria bassiana* and *Metarhizium anisopliae* isolates for management of adult *Spodoptera frugiperda*. Southwest. Entomol..

[21] Ramanujam B., Poornesha B., Shylesha A.N. (2020). Effect of entomopathogenic fungi against invasive pest *Spodoptera frugiperda* (J. E. Smith) (Lepidoptera: Noctuidae) in maize. Egypt. J. Biol. Pest Control..

[22] Ramos Y., Taibo A.D., Jiménez J.A., Portal O. (2020). Endophytic establishment of *Beauveria bassiana* and *Metarhizium anisopliae* in maize plants and its effect against *Spodoptera frugiperda* (J. E. Smith) (Lepidoptera: Noctuidae) larvae. Egypt. J. Biol. Pest Control..

[23] Hu G., Leger R.J.S. (2002). Field studies using a recombinant mycoinsecticide (*Metarhizium anisopliae*) reveal that it is rhizosphere Competent. Appl. Environ. Microbiol..

[24] Vega F.E. (2018). The use of fungal entomopathogens as endophytes in biological control: A review. Mycologia.

[25] White J.F., Kingsley K.L., Zhang Q., Verma R., Obi N., Dvinskikh S., Elmore M.T., Verma S.K., Gond S.K., Kowalski K.P. (2019). Review: Endophytic microbes and their potential applications in crop management. Pest Manag. Sci..

[26] Ahmad I., Jiménez-Gasco M.D.M., Barbercheck M.E. (2020). The role of endophytic insect-pathogenic fungi in biotic stress management.

[27] Ahmad I., Jiménez-Gasco M.D.M., Luthe D.S., Shakeel S.N., Barbercheck M.E. (2020). Endophytic *Metarhizium robertsii* promotes maize growth, suppresses insect growth, and alters plant defense gene expression. Biol. Control..

[28] Dutta P., Kaushik H., Bhawmick P., Puzari K.C., Hazarika G.N. (2015). *Metarhizium anisopliae* as endophyte has the ability of plant growth enhancement. Int. J. Curr. Res..

[29] García E.J., Posadas B.J., Perticari A., Lecuona R.E. (2011). *Metarhizium anisopliae* (Metschnikoff) Sorokin promotes growth and has endophytic activity in tomato plants. Adv. Biol. Res..

[30] Golo P.S., Gardner D.R., Grilley M.M., Takemoto J.Y., Krasnoff S.B., Pires M.S., Fernandes E., Bittencourt V.R.E.P., Roberts D.W. (2014). Production of destruxins from *Metarhizium* spp. fungi in artificial medium and in endophytically colonized cowpea plants. PLoS ONE.

[31] Kaushik H., Dutta P., Himadri K., Pranab D. (2016). Establishment of *Metarhizium anisopliae*, an entomopathogen as endophyte for biological control in tea. Res. Crop..

[32] Mantzoukas S., Chondrogiannis C., Grammatikopoulos G. (2015). Effects of three endophytic entomopathogens on sweet sorghum and on the larvae of the stalk borer *Sesamia nonagrioides*. Entomol. Exp. Appl..

[33] Behie S.W., Jones S.J., Bidochka M.J. (2015). Plant tissue localization of the endophytic insect pathogenic fungi *Metarhizium* and *Beauveria*. Fungal Ecol..

[34] Liao X., O’Brien T.R., Fang W., Leger R.J.S. (2014). The plant beneficial effects of *Metarhizium* species correlate with their association with roots. Appl. Microbiol. Biotechnol..

[35] Ríos-Moreno A., Garrido-Jurado I., Resquín-Romero G., Arroyo-Manzanares N., Arce L., Quesada-Moraga E. (2016). Destruxin A production by Metarhizium brunneum strains during transient endophytic colonization of *Solanum tuberosum*. Biocontrol Sci. Technol..

[36] Cruz-Avalos A.M., Bivián-Hernández M.D.L.Á., Ibarra J.E., Del Rincón-Castro M.C. (2019). High virulence of Mexican entomopathogenic fungi against fall armyworm (Lepidoptera: Noctuidae). J. Econ. Entomol..

[37] Grijalba E.P., Espinel C., Cuartas P.E., Chaparro M.L., Villamizar L.F. (2018). *Metarhizium rileyi* biopesticide to control *Spodoptera frugiperda*: Stability and insecticidal activity under glasshouse conditions. Fungal Biol..

[38] Hernandez-Trejo A., Estrada-Drouaillet B., López-Santillán J.A., Rios-Velasco C., Rodríguez-Herrera R., Osorio-Hernández E. (2019). Effects of native entomopathogenic fungal strains and neem extract on *Spodoptera frugiperda* on Maize. Southwest. Entomol..

[39] Akutse K.S., Kimemia J.W., Ekesi S., Khamis F.M., Ombura O.L., Subramanian S. (2019). Ovicidal effects of entomopathogenic fungal isolates on the invasive Fall armyworm *Spodoptera frugiperda* (Lepidoptera: Noctuidae). J. Appl. Entomol..

[40] de Lira A.C., Mascarin G.M., Delalibera I. (2020). Microsclerotia production of *Metarhizium* spp. for dual role as plant biostimulant and control of *Spodoptera frugiperda* through corn seed coating. Fungal Biol..

[41] Mwamburi L.A. (2021). Endophytic fungi, *Beauveria bassiana* and *Metarhizium anisopliae*, confer control of the fall armyworm, *Spodoptera frugiperda* (J. E. Smith) (Lepidoptera: Noctuidae), in two tomato varieties. Egypt. J. Biol. Pest Control..

[42] Randhawa P.K., Mullen C., Barbercheck M. (2018). Plant identity, but not diversity, and agroecosystem characteristics affect the occurrence of *M. robertsii* in an organic cropping system. Biol. Control..

[43] Zimmermann G. (1986). The ‘Galleria bait method’ for detection of entomopathogenic fungi in soil. J. Appl. Entomol..

[44] Bischoff J.F., Rehner S.A., Humber R.A. (2009). A multilocus phylogeny of the *Metarhizium anisopliae* lineage. Mycologia.

[45] Kepler R.M., Ugine T.A., Maul J.E., Cavigelli M.A., Rehner S.A. (2015). Community composition and population genetics of insect pathogenic fungi in the genus *Metarhizium* from soils of a long-term agricultural research system. Environ. Microbiol..

[46] Parsa S., Ortiz V., Vega F.E. (2013). Establishing fungal entomopathogens as endophytes: Towards endophytic biological control. J. Vis. Exp..

[47] Jager G., Van Der Boon J., Rauw G. (1969). The influence of soil steaming on some properties of the soil and on the growth and heading of winter glasshouse lettuce. I. Changes in chemical and physical properties. Neth. J. Agric. Sci..

[48] Peiffer M., Felton G.W. (2005). The host plant as a factor in the synthesis and secretion of salivary glucose oxidase in larval *Helicoverpa zea*. Arch. Insect Biochem. Physiol..

[49] Perkins W.D. (1979). Laboratory rearing of the fall armyworm. Fla. Entomol..

[50] Chuang W.-P., Ray S., Acevedo F.E., Peiffer M., Felton G.W., Luthe D.S. (2014). Herbivore cues from the fall armyworm (*Spodoptera frugiperda*) larvae trigger direct defenses in maize. Mol. Plant Microbe Interact..

[51] Hoffmann W.A., Poorter H. (2002). Avoiding bias in calculations of relative growth rate. Ann. Bot..

[52] Henniges-Janssen K., Heckel D.G., Groot A.T. (2014). Preference of diamondback moth larvae for novel and original host plant after host range expansion. Insects.

[53] Ives A.R. (2015). For testing the significance of regression coefficients, go ahead and log-transform count data. Methods Ecol. Evol..

[54] Sergaki C., Lagunas B., Lidbury I., Gifford M.L., Schäfer P. (2018). Challenges and approaches in microbiome esearch: From fundamental to applied. Front. Plant Sci..

[55] Razinger J., Lutz M., Schroers H.-J., Urek G., Grunder J. (2014). Evaluation of insect associated and plant growth promoting fungi in the control of cabbage root flies. J. Econ. Entomol..

[56] Sasan R.K., Bidochka M.J. (2012). The insect-pathogenic fungus *Metarhizium robertsii* (Clavicipitaceae) is also an endophyte that stimulates plant root development. Am. J. Bot..

[57] Clay K., Cheplick G.P. (1989). Effect of ergot alkaloids from fungal endophyte-infected grasses on fall armyworm (*Spodoptera frugiperda*). J. Chem. Ecol..

[58] Resquín-Romero G., Garrido-Jurado I., Delso C., Rios-Moreno A., Quesada-Moraga E.J. (2016). Transient endophytic colonizations of plants improve the outcome of foliar applications of mycoinsecticides against chewing insects. J. Invertebr. Pathol..

[59] Israni B., Wouters F.C., Luck K., Seibel E., Ahn S.-J., Paetz C., Reinert M., Vogel H., Erb M., Heckel D.G. (2020). The fall armyworm *Spodoptera frugiperda* utilizes specific UDP-glycosyltransferases to inactivate maize defensive benzoxazinoids. Front. Physiol..

[60] Carvalho I., Erdmann L.L., Machado L.L., Rosa A.P.S.A., Zotti M.J., Neitzke C.G. (2018). Metabolic resistance in the fall armyworm: An overview. J. Agric. Sci..

[61] Chikate Y.R., Tamhane V., Joshi R.S., Gupta V.S., Giri A.P. (2013). Differential protease activity augments polyphagy in *Helicoverpa armigera*. Insect Mol. Biol..

[62] Giraudo M., Hilliou F., Fricaux T., Audant P., Feyereisen R., LE Goff G. (2014). Cytochrome P450s from the fall armyworm (*Spodoptera frugiperda*): Responses to plant allelochemicals and pesticides. Insect Mol. Biol..

[63] Li J., Zhang C., Xu X., Wang J., Yu H., Lai R., Gong W. (2007). Trypsin inhibitory loop is an excellent lead structure to design serine protease inhibitors and antimicrobial peptides. FASEB J..

[64] Després L., David J.-P., Gallet C. (2007). The evolutionary ecology of insect resistance to plant chemicals. Trends Ecol. Evol..

[65] Appel H.M. (1994). The chewing herbivore gut lumen: Physicochemical conditions and their impact on plant nutrients, allelo-chemicals, and insect pathogens. Insect-Plant Interactions.

[66] Gimenez S., Abdelgaffar H., Le Goff G., Hilliou F., Blanco C.A., Hänniger S., Bretaudeau A., Legeai F., Nègre N., Jurat-Fuentes J.L. (2020). Adaptation by copy number variation increases insecticide resistance in the fall armyworm. Commun. Biol..

[67] De Bruyn L., Scheirs J., Verhagen R. (2002). Nutrient stress, host plant quality and herbivore performance of a leaf-mining fly on grass. Oecologia.

[68] Larsson S. (1989). Stressful times for the plant stress: Insect performance hypothesis. Oikos.

[69] Krell V., Jakobs-Schoenwandt D., Vidal S., Patel A.V. (2018). Encapsulation of *Metarhizium brunneum* enhances endophytism in tomato plants. Biol. Control..

[70] Niemeyer H.M. (2009). Hydroxamic acids derived from 2-Hydroxy-2H-1,4-Benzoxazin-3(4H)-one: Key defense chemicals of cereals. J. Agric. Food Chem..

[71] Shoresh M., Harman G.E., Mastouri F. (2010). Induced systemic resistance and plant responses to fungal biocontrol agents. Annu. Rev. Phytopathol..

[72] Lòpez-Fernàndez S., Compant S., Vrhovsek U., Bianchedi P.L., Sessitsch A., Pertot I., Campisano A. (2015). Grapevine colonization by endophytic bacteria shifts secondary metabolism and suggests activation of defense pathways. Plant Soil.

[73] Kunkel B.N., Brooks D.M. (2002). Cross talk between signaling pathways in pathogen defense. Curr. Opin. Plant Biol..

[74] Spoel S.H., Koornneef A., Claessens S.M.C., Korzelius J.P., Van Pelt J.A., Mueller M.J., Buchala A.J., Métraux J.-P., Brown R., Kazan K. (2003). NPR1 modulates cross-talk between salicylate- and jasmonate-dependent defense pathways through a novel function in the cytosol. Plant Cell.

[75] Clifton E.H., Jaronski S.T., Coates B.S., Hodgson E.W., Gassmann A.J. (2018). Effects of endophytic entomopathogenic fungi on soybean aphid and identification of *Metarhizium* isolates from agricultural fields. PLoS ONE.

[76] Rasool S., Vidkjær N.H., Hooshmand K., Jensen B., Fomsgaard I.S., Meyling N.V. (2021). Seed inoculations with entomopathogenic fungi affect aphid populations coinciding with modulation of plant secondary metabolite profiles across plant families. New Phytol..

[77] Cachapa J.C., Meyling N.V., Burow M., Hauser T.P. (2021). Induction and priming of plant defense by root-associated insect-pathogenic fungi. J. Chem. Ecol..

[78] Erb M., Meldau S., Howe G.A. (2012). Role of phytohormones in insect-specific plant reactions. Trends Plant Sci..

[79] Gichuhi J., Sevgan S., Khamis F., Berg J.V.D., Du Plessis H., Ekesi S., Herren J. (2020). Diversity of fall armyworm, *Spodoptera frugiperda* and their gut bacterial community in Kenya. PeerJ.

[80] Lezama Gutierrez R., Alatorre Rosas R., Bojalil Jaber L.F., Molina Ochoa J., Arenas Vargas M., Gonzalez Ramirez M., Rebolledo Dominguez O. (1996). Virulence of five entomopathogenic fungi (Hyphomycetes) against *Spodoptera frugiperda* (Lepidop-tera: Noctuidae) eggs and neonate larvae. Vedalia Rev. Int. Control Biol..

[81] Schmelz E.A., Alborn H.T., Tumlinson J.H. (2001). The influence of intact-plant and excised-leaf bioassay designs on volicitin- and jasmonic acid-induced sesquiterpene volatile release in *Zea mays*. Planta.

[82] Acevedo F.E., Peiffer M., Tan C.-W., Stanley B.A., Stanley A., Wang J., Jones A.G., Hoover K., Rosa C., Luthe D. (2017). Fall armyworm-associated gut bacteria modulate plant defense responses. Mol. Plant Microbe Interact..

[83] Santiago R., Cao A., Butrón A., López-Malvar A., Rodríguez V.M., Sandoya G.V., Malvar R.A. (2017). Defensive changes in maize leaves induced by feeding of Mediterranean corn borer larvae. BMC Plant Biol..

[84] War A.R., Paulraj M.G., Ahmad T., Buhroo A.A., Hussain B., Ignacimuthu S., Sharma H.C. (2012). Mechanisms of plant defense against insect herbivores. Plant Signal. Behav..

[85] Wu J., Baldwin I.T. (2010). New insights into plant responses to the attack from insect herbivores. Annu. Rev. Genet..

[86] Cook D., Gardner D.R., Welch K.D., Roper J.M., Ralphs M.H., Green B.T. (2009). Quantitative PCR method to measure the fungal endophyte in locoweeds. J. Agric. Food Chem..

[87] Jaber L.R., Vidal S. (2010). Fungal endophyte negative effects on herbivory are enhanced on intact plants and maintained in a subsequent generation. Ecol. Entomol..

